# Supplementary material for: Thiourea Derivatives, Simple in Structure but Efficient Enzyme Inhibitors and Mercury Sensors
Source: Molecules. 2021 Jul 27;26(15):4506. doi: 10.3390/molecules26154506 (PMC8347686; doi:10.3390/molecules26154506)
Supplement: Supplementary file 1 [file molecules-26-04506-s001.zip › molecules-1260380-supplementary.pdf]

molecules

MDPI

Supplementary Materials

Thiourea Derivatives, Simple in Structure and Efficient Enzyme Inhibitorsand Mercury Sensors

Faizan Ur Rahman, Maryam Bibi, Ezzat Khan, Abdul Bari Shah, Mian Muhammad, Muhammad Nawaz Tahir, Adnan Shahzad, Farhat Ullah, Muhammad Zahoor, Salman Alamery and Gaber El-Saber Batiha

Supplementary details

1. Synthesis of compounds 1 to 6

1.1. 1-cyclohexyl-3-isobutylthiourea, **1**

Compound **1** was synthesized by dropwise addition of cyclohexyl isothiocyante (1 mL, 7.05 mmol) to isobutyl amine (0.7 mL, 7.05 mmol) in analytical grade acetone. The reaction mixture was allowed to stir over night at room temperature. The reaction led to the formation of precipitates and the progress was monitored by TLC. Solid material was separated from the mother liquor, washed with petroleum ether for removal of unreacted compounds and possible impurities. The solid was re-dissolves in ethanol and kept for slow evaporation. Spectroscopic data were collected and structure of the desired compound was deduced.

Yield = 70 %; Molecular formula = $C_{11}H_{22}N_2S$; m.p. = 102-105°C; **FT-IR** (ATR): ν (cm^{-1}) = 3431br (NH), 3214br (N-H), 2930s, 2850w (CH), 1617 (C=S), 1379 (NCN), 1448 (CS_{asy}); **^{1}H-NMR** (300 MHz, DMSO-*d*$_6$) δ (ppm)= 0.85 (d, *J*(^{1}H,^{1}H) = 6.7 Hz, 6H, Me), 1.16, 1.25, 1.53, 1.63, 1.82 (overlapping multiplets of aliphatic protons), 3.18 (br, 1H, NH), 3.94 (br, 1H, NH). **^{13}C-NMR** (75 MHz, DMSO-*d*$_6$) δ (ppm) = 20.1 (2C), 24.5 (2C), 25.1 (1C), 27.7 (1C), 32.3 (2C), 50.9 (1C), 51.6 (1C), 181.3 (C=S).

1.2. 1-cyclohexyl-3-(tert-butyl)thiourea, **2**

In the similar manner as described above compound **2** was prepared by mixing *tert*-butylamine (1 mL, 9.5 mmol) with a solution of cyclohexyl isothiocyanate (1.35 mL, 9.5 mmol) in dry acetone. Compound **2** was obtained as a colorless solid in ethanol.

Yield = 68 %; Molecular formula = $C_{11}H_{22}N_2S$; m.p.= 138-140°C; **FT-IR** (ATR): ν(cm^{-1}) = 3421br, 3367br (N-H), 3050w, 2927w (C-H), 1636s (C=S), 1385 (NCN), 1544 (CS_{asy}); **^{1}H-NMR**(300 MHz DMSO *d*$_6$) δ (ppm) = 1.03 (m, 2H, CH_2), 1.20 (m, 4H, CH_2), 1.61 (m, 4H, CH_2), 1.41 (s, 9H, Me), 1.83 (m, 1H, CH), 2.50 (p, 1H, CH), 3.16 (d, 1H, NH), 4.08 (br, 1H, NH); **^{13}C-NMR** (75 MHz, DMSO-*d*$_6$) δ (ppm) = 24.4, 25.2, 29.0, 32.3, 50.6, 52.0, 180.2 (C=S).

1.3. 1-cyclohexyl-3-(3-chlorophenyl)thiourea, **3**

A known amount of 3-chloroaniline (0.5 mL, 4.7 mmol) in 15 mL analytical grade acetone and equimolar amount of cyclohexylisothiocyante (0.6 mL, 4.7 mmol) was reacted together following the same method. Colorless crystals of **3** were obtained in EtOH in few days, were separated from the mother liquor, FT-IR and NMR data were collected and suitable crystal of the compound was mounted for X-ray data collection and structure confirmation.

Yield = 66 %; Molecular formula = $C_{13}H_{17}ClN_2S$, m.p. = 115-120°C, **FT-IR** (ATR): ν(cm^{-1}) 3295br, 3206br (N-H), 3060w (Ar, C-H), 2993w, 2849w (C-H), 1700s (C=S), 1534s

(NCN), 1435s (CS_{asy}); ^{1}**H-NMR** (300 MHz, DMSO-d_6) δ (ppm) = 1.26 (m, 4H, CH_2), 1.67 (m, 4H, CH_2), 1.92 (m, 2H, CH_2), 1.99 (m, 1H, CH), 4.17 (br, 1H, NH), 7.10, 7.26, 7.31, 7.82 (m, m, m, m, 4H, CH, Ph), 9.45 (br, 1H, NH); ^{13}**C-NMR** (DMSO-d_6) δ (ppm) = 25.0, 31.9, 39.8, 52.5, 121.0, 122.1, 123.5, 130.0, 132.9, 141.8, 179.5 (C=S)

1.4. 1-phenyl-3-(1,1-dibutyl)thiourea, 4

Phenyl isothiocynate(1mL,8.3mmol)anddibutylamine (0.88 mL, 8.3 mmol) in 20 mL acetone were treated as discussed above. Colorless crystals of the desired compound **4** were obtained in the same solvent at ambient temperature.

Yield = 95%; Molecular formula = $C_{15}H_{24}N_2S$, m.p. = 85-87°C; **FT-IR** (ATR 400-4000 cm^{-1}) ν (cm^{-1}) = 3700br (N-H), 2335w (C-H), 1529s (C=C), 1452s (NCN), 1205s (CS_{asy}); ^{1}**H-NMR** (300 MHz, $CDCl_3$), δ (ppm) = 0.96 (t, 6H, Me), 1.37 (m, 4H, CH_2), 1.69 (pent, 4H, CH_2), 3.65 (t, 4H, CH_2N), 7.05 (br, 1H, NH), 7.18, 7.31 (m, m, 5H, Ph); ^{13}**C-NMR** (75 MHz, $CDCl_3$) δ (ppm) = 13.7, 20.1, 29.4, 51.4, 125.5, 125.6, 128.5, 139.8, 181.0 (C=S).

1.5. 1-Phenyl-3-(2-chlorophenyl)thiourea, 5

In the similar manner as described above by reacting 2-chloroanaline (0.87 mL, 8.3 mmol) with phenylisothiocyanate (1 mL, 8.3 mmol), the product **5** was obtained as colorless crystalline solid by recrystallization from ethanol. In the whole reaction process the reaction was monitored by TLC until single spot product was formed.

Yield = 69 %; Molecular formula = $C_{13}H_{11}ClN_2S$; m.p. = 90-92°C; **FT-IR** (ATR) :ν(cm^{-1}) = 3700br (NH), 2335w (C-H), 1528s (C=S), 1452s (NCN), 1442s (CS_{asy}); ^{1}**H-NMR** (DMSO-d_6) δ (ppm) = 7.16, 7.23-7.29, 7.32-7.39, 7.53, 7.63 (m, m, m, m, m, 9H, Aromatic protons), 9.43 (br, 1H, NH), 10.02 (br, 1H, NH); ^{13}**C-NMR** (DMSO-d_6) δ (ppm) = 123.8, 124.7, 127.2, 127.5, 128.6, 129.4, 129.8, 130.0, 136.4, 139.2, 180.3 (C=S).

1.6. 1-phenyl-3-(4-chlorophenyl)thiourea, 6

The derivative **6**, was obtained as colorless solid by treating 4-chloroaniline (0.8 mL, 8.3 mmol) and phenylisothiocyanate (1.0 mL, 8.3 mmol) in the same manner as reported in literature [1]. Crystals were obtained in ethanol at room temperature by slow evaporation method.

Yield = 70 %; Molecular formula = $C_{13}H_{11}ClN_2S$; m. p. = 95-99°C; **FT-IR** (ATR): ν(cm^{-1}) = 3436-3100br (N-H), 3000w (C-H), 1637s (C=S), 1549s (NCN), 1490s (CS_{asy}); ^{1}**H-NMR** (DMSO-d_6) δ (ppm) = 7.14, 7.37, 7.52 (m, m, m, 10H, Aromatic protons, NH), 9.88 (br, 1H, NH); ^{13}**C-NMR** (DMSO-d_6) δ (ppm) = 123.8, 124.6, 125.3, 128.3, 128.5, 138.5, 139.3, 179.3 (C=S).

2. FT-IR spectra of compounds 1–6

The FT-IR of all synthetic compounds were measured in the range 4000-400 cm^{-1}. In all spectra two strong absorption peaks with typical broader shape were found in the range 3465-3100 cm^{-1}, correspond to N-H bond stretching with extensive H-bonding environment. Sharp and intense peaks were noted in case of **1** and **3** while broad for other compounds. In case of all compounds the aromatic and aliphatic C-H peaks were well resolved, while overlapping of aromatic C-H bonds with N-H was noticed where both reactants are aromatic in nature (**5**, **6)**. From 2993-2839 cm^{-1} sharp peaks were noticed which correspond to C-H saturated bonds in **1**, **2** and **3**. While in case of **5** and **6**a couple of peaks 3185-3001 cm^{-1} along with N-H stretch were noticed showing aromatic C-H bonds. Similarly, from the region 1700-1549 cm^{-1} the aromatic C=C and C=S double bonds are expected[2]. From FT-IR spectra it is very clear that extensive Hydrogen bonding is present in **3** and **4** which in other words show intermolecular interactions in the

region (3833-3742 cm^{-1}). The observation from FT-IR data is in line with solid state X-ray diffraction data, *vide infra*.

3. *NMRspectra of compounds* **1–6**

The ^{1}H- and^{13}C-NMR of all compounds were recorded on Bruker AM 300 MHz spectrometer. In the ^{1}H-NMRspectra, all the aliphatic and aromatic protons appeared in their characteristic and expected region, 0.8-3.4 ppm and 7.0-7.9 ppm, respectively[2]. The NMR data analysis of all compounds reveal that the reaction is straightforward and no side reactions were observed. As each compound contains two types of N-H protons with different environment (except **4**), and accordingly two signals were observed for each of them. The N-H proton close to aromatic ring was observed in up-field region while the other appeared in down-field region.

From ^{13}C-NMR spectra of compounds **1-6,** it is evidenced that all the aliphatic and aromatic carbons appeared below 50 ppm and 120-140 ppm, respectively. The chemical shift corresponding to C=S bond was noticed in the region of ≈180 ppm, the presence of this carbon in the spectra of compounds **1-6** provides enough justification regarding the formation and presence of thiourea derivatives. Other carbon atoms belong to molecules of respective compounds appeared in their characteristic regions.

Table S1. UV-Visible spectroscopic data of compounds **1–3**, **5** and **6.**

Compound	Concentration (μg mL^{-1})	Scan range (nm)	λ_{max} (nm)
**1**		200-400	296
**2**		200-400	294
**3**	2	200-400	298
**5**		200-400	294
**6**		200-400	295

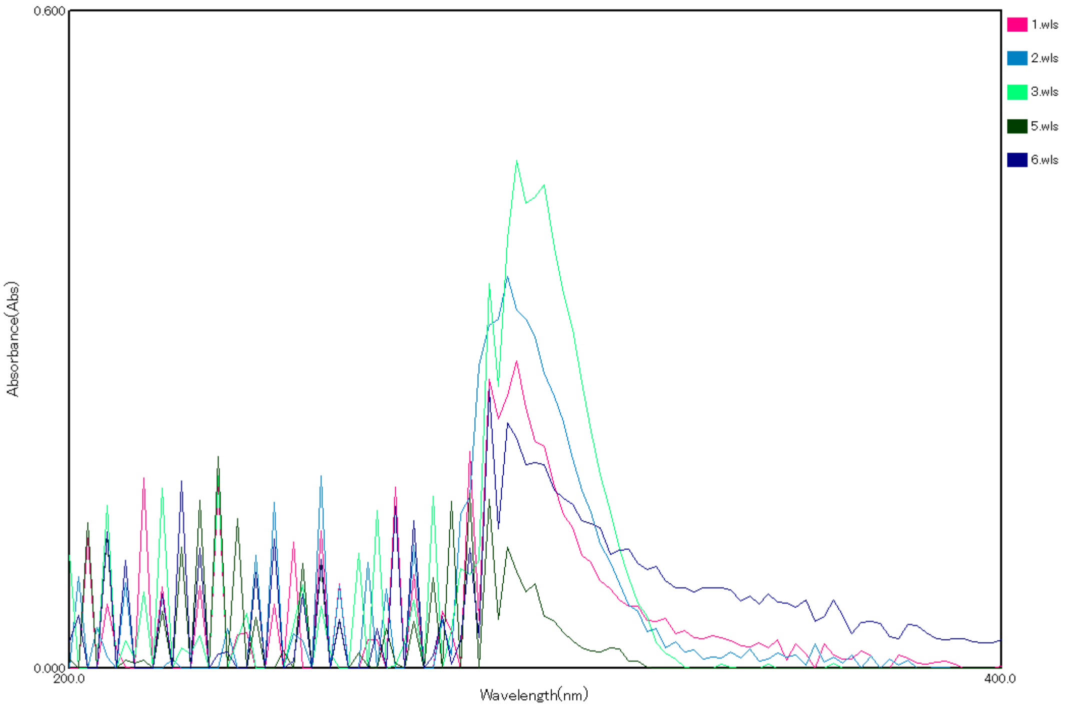

Figure S1. UV absorption spectra of compounds **1–3, 5** and **6.**

References

[1] P. Thanigaimalai: K.-C. Lee, V.K. Sharma, C. Joo, W.-J. Cho, E. Roh, Y. Kim, S.-H. Jung, Bioorganic & Medicinal Chemistry Letters 21 (2011) 6824-6828.
[2] F.U. Rahman, M. Bibi, A.A. Altaf, M.N. Tahir, F. Ullah, E. Khan, Journal of Molecular Structure 1211 (2020) 128096.
